# Composite Chitosan/Agarose Ferrogels for Potential Applications in Magnetic Hyperthermia

**DOI:** 10.3390/gels1010069

**Published:** 2015-07-09

**Authors:** Vanessa Zamora-Mora, Paula I.P. Soares, Coro Echeverria, Rebeca Hernández, Carmen Mijangos, Rolando Barbucci

**Affiliations:** 1 Instituto de Ciencia y Tecnología de Polímeros (ICTP-CSIC) c/ Juan de la Cierva 3, 28006 Madrid, Spain; E-Mail: vzamora4@gmail.com; 2 CENIMAT/I3N, Departamento de Ciência dos Materiais, Faculdade de Ciências e Tecnologia, FCT, Universidade Nova de Lisboa, 2829-516 Caparica, Portugal; E-Mails: paulaipsoares@gmail.com (P.I.P.S.), coro@fct.unl.pt (C.E.)

**Keywords:** chitosan, agarose, magnetic nanoparticles, magnetic hyperthermia

## Abstract

Composite ferrogels were obtained by encapsulation of magnetic nanoparticles at two different concentrations (2.0 and 5.0 % *w*/*v*) within mixed agarose/chitosan hydrogels having different concentrations of agarose (1.0, 1.5 and 2.0% (*w*/*v*)) and a fixed concentration of chitosan (0.5% (*w*/*v*)). The morphological characterization carried out by scanning electron microscopy showed that dried composite ferrogels present pore sizes in the micrometer range. Thermogravimetric measurements showed that ferrogels present higher degradation temperatures than blank chitosan/agarose hydrogels without magnetic nanoparticles. In addition, measurements of the elastic moduli of the composite ferrogels evidenced that the presence of magnetic nanoparticles in the starting aqueous solutions prevents to some extent the agarose gelation achieved by simply cooling chitosan/agarose aqueous solutions. Finally, it is shown that composite chitosan/agarose ferrogels are able to heat in response to the application of an alternating magnetic field so that they can be considered as potential biomaterials to be employed in magnetic hyperthermia treatments.

## 1. Introduction 

Hydrogels are defined as three-dimensional polymer networks that are able to retain a large amount of water in their swollen state [1]. Specifically, hydrogels obtained from natural polymers are currently the focus of considerable scientific research for the development of biomedical applications due to their inherent biocompatibility, biodegradability and also because they are susceptible to enzymatic degradation [2,3,4]. An example of natural polymer commonly employed for the preparation of hydrogels is chitosan. Chitosan is soluble in certain acidic aqueous solutions due to the protonation of the primary amine groups. Due to the pH-responsiveness and inherent biocompatibility, chitosan gels are attractive for biomedical applications [5,6,7] and specially, as materials for controlled drug delivery [8,9,10,11]. 

Chitosan hydrogels can be obtained either by physical associations, such as secondary forces (hydrogen, ionic, or hydrophobic bonding) and physical entanglements and by covalent crosslinking [2]. Chitosan aqueous solutions can become a gel in alkaline solutions without any chemical cross-linkers due to the fact that positive charges of chitosan molecules are neutralized resulting in coacervation-phase inversion with gel formation. This property can be employed for the *in situ* synthesis of iron oxide nanoparticles employing chitosan gels as templates. In a first step, iron cations are solubilized in an acid aqueous solution of chitosan and, as a second step, the oxidation in alkaline solution leads to the simultaneous formation of magnetic nanoparticles (magnetite or maghemite) and chitosan gelation [12]. Another method to prepare magnetic chitosan hydrogels consists in the encapsulation of preformed magnetic nanoparticles into a chitosan matrix [13,14]. These hybrid chitosan hydrogels, also known as ferrogels, can be implemented as materials for biomedical applications, *i.e*., materials employed in magnetic hyperthermia, that is, able to heat up target tumors remotely through an external magnetic field [15,16,17].

In order to broaden the applications of chitosan hydrogels, researchers have developed composite hydrogels, where chitosan is blended with other biopolymers, e.g. collagen [18], gelatin [19] and agarose [20]. In particular, agarose, an alternating copolymer found in some seaweeds consisting of 1,4-linked 3,6-anhydro-α-l-galactose and 1,3-linked β-d-galactose derivatives is a neutral polysaccharide that forms thermoreversible gels upon cooling agarose aqueous solutions. The biocompatibility of agarose hydrogels and the mild conditions required for its gelation, make the composite hydrogel suitable for biomedical applications, in particular, for cell culture and sustained drug delivery [21]. In addition, mixing of agarose with chitosan can be used to improve the mechanical properties of agarose, [22,23] or properties of adhesion of cells [23]. Recently, mixtures of chitosan and agarose have been prepared by a suspension cross-linking method [24], water-in-oil emulsion technique [25] or via electrospinning [26]. 

The gelation of agarose in mixed aqueous solutions of chitosan and agarose leads to the formation of transparent and mechanically stable hydrogels in which chitosan chains remain embedded into the agarose matrix. In this way, the pH and temperature response of the resulting composite gels is retained [27]. The aim of this work is to collective evidence of a facile and simple approach to obtain biodegradable and biocompatible ferrogels from natural sources and their ability to respond to an applied external magnetic field, as an indication of the feasibility to be employed as potential materials for magnetic hyperthermia. In this work, we employ the same methodology previously used to obtain Chitosan/Agarose (Chi/Aga) composite ferrogels, without chemical crosslinking, through encapsulation of magnetic nanoparticles dispersed as an aqueous ferrofluid at two different concentrations and we report the preparation method, the morphology, the mechanical properties as a function of temperature, and the Specific Power Absorption (SPA) measurements. 

## 2. Results and Discussion 

### 2.1. Morphological Study

**Figure 1:**
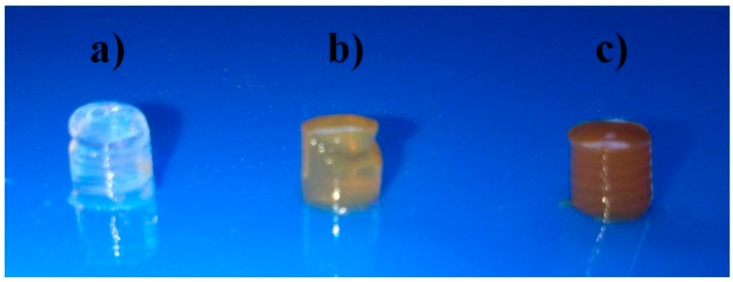
Photographs of (**a**) Chi/Aga-1.5 hydrogel and its ferrogels (**b**) Chi/Aga-1.5+Fe2% and (**c**) Chi/Aga-1.5+Fe5%.

Figure 1 shows the macroscopic appearance of a composite Chi/Aga-1.5 hydrogel and its corresponding ferrogels loaded with two different concentrations of ferrofluid (2.0% and 5.0% (*w*/*v*)). Chi/Aga blank hydrogels are transparent in the hydrated state, which suggest uniform distribution of chitosan in the host agarose matrix, without noticeable phase separation, as previously reported [27]. On the other hand, Chi/Aga ferrogels (Chi/Aga+Fe) present the characteristic brown color corresponding to the ferrofluid, which gives evidence to the encapsulation of magnetic nanoparticles within the polymer hydrogels. 

**Figure 2:**
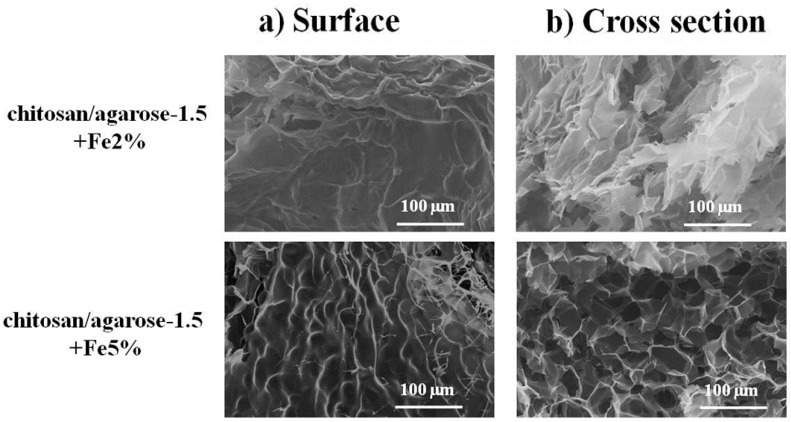
SEM images corresponding to the (**a**) surface and (**b**) cross section of Chi/Aga-1.5 ferrogels loaded with magnetic nanoparticles at 2.0% and 5.0% (*w*/*v*).

The morphology of the dried ferrogels, Chi/Aga-1.5+Fe2% and Chi/Aga-1.5+Fe5% was further analyzed through SEM. The results corresponding to the surface and cross section are shown in Figure 2. As can be observed, both samples present a rough surface. The pore size determined from the images corresponding to the cross section was 37 ± 5 µm for Chi/Aga-1.5+Fe2% and 51 ± 9 µm for Chi/Aga-1.5+Fe5%.

### 2.2. Thermal Stability

**Figure 3:**
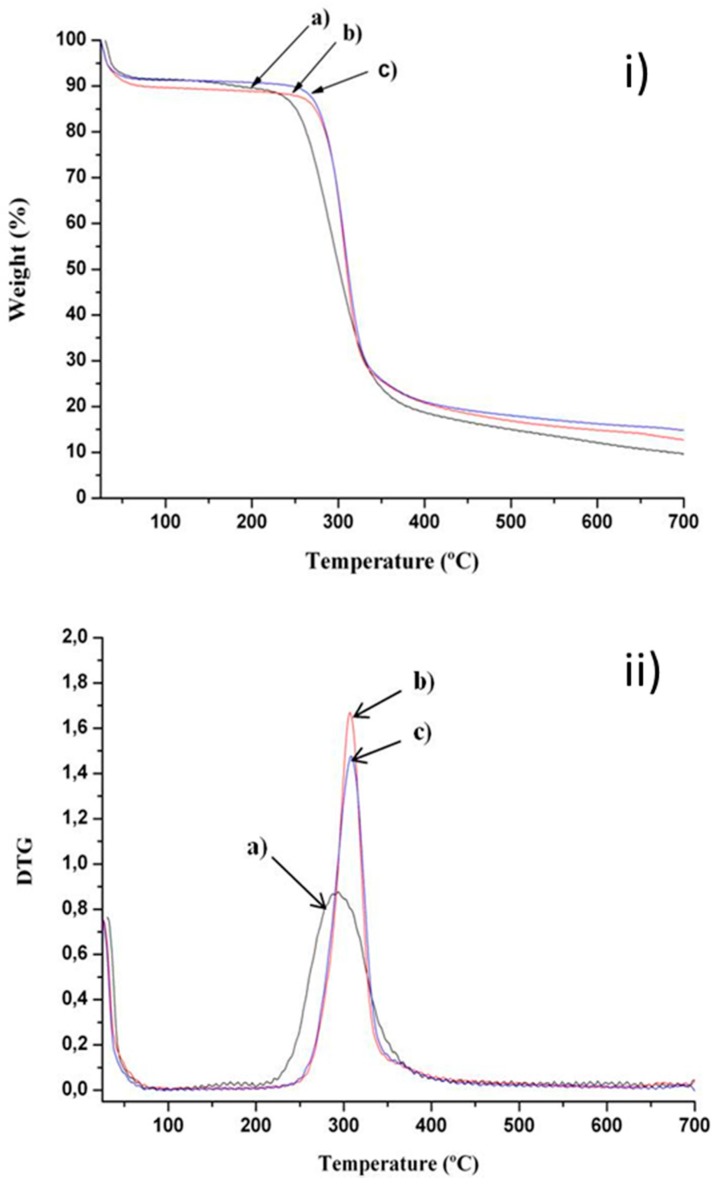
(**i**) Thermal degradation and (**ii**) differential thermogravimetric curves of (a) Chi/Aga-2 (black), (b) Chi/Aga-2+Fe2% (red) and (c) Chi/Aga-2+Fe5% (blue).

The thermal stability of dried composite Chi/Aga hydrogels and ferrogels was determined through thermogravimetric analysis. Representative results corresponding to samples Chi/Aga-2, Chi/Aga-2+Fe2% and Chi/Aga+Fe5% are shown in Figure 3. The three samples presented a first weight loss (~9%) between 25 and 80 °C, which is attributed to the evaporation of superficial water. Then, the second weight loss stage in the range of 150–500 °C is due to the decomposition of the organic chains of polymers, chitosan [28] and agarose [29] with similar weight losses, ~75%. According to the derivative of TGA (Figure 3b), the corresponding degradation temperatures for Chi/Aga-2, Chi/Aga-2+Fe2% and Chi/Aga-2+Fe5% are 294, 307 and 308 °C, respectively. The difference among degradations temperatures of composite ferrogels could be attributed to the establishment of interactions among the polymer matrix and the magnetic nanoparticles, as previously reported [12] for other nanocomposite polymer materials. 

The residues obtained for Chi/Aga-2, Chi/Aga-2+Fe2% and Chi/Aga-2+Fe5% at 600 °C are 12%, 15% and 16%, respectively. The magnetite content (Fe_3_O_4_ % *w*/*v*) in composite chitosan/agarose ferrogels can be determined by subtracting the residue corresponding to the blank hydrogel (Chit/Aga-2), being 3 and 4% (*w*/*w*) for Chi/Aga-2+Fe2% and Chi/Aga-2+Fe5%, respectively. 

### 2.3. Viscoelastic Properties

**Figure 4:**
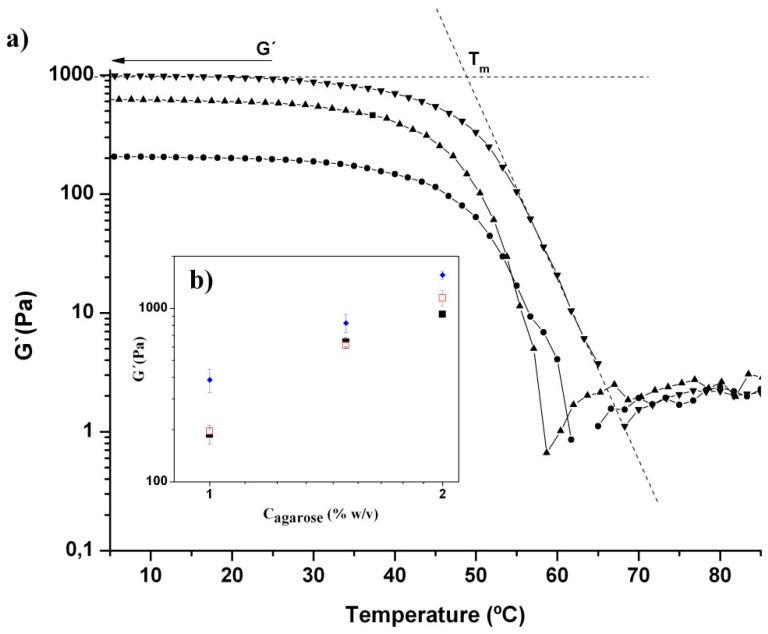
(**a**) Elastic modulus, G′, plotted as a function of temperature for (●) Chi/Aga-1+Fe5%, (▲) Chi/Aga-1.5+Fe5% and (▼) Chi/Aga-2+Fe5%. Dashed lines show the determination of the melting temperature, *T*_m_, of the gel. (**b**) Inset image corresponds to the variation in G´ as a function of agarose concentration for Chi/Aga ferrogels loaded with different concentrations of ferrofluid ((♦) 0% (*w*/*v*); (■) 2% (*w*/*v*) and (□) 5% *w*/*v*)

Figure 4 shows the variation in elastic modulus, G′, with temperature of Chi/Aga ferrogels loaded with the same concentration of ferrofluid (5% (*w*/*v*)) at different concentrations of agarose. As can be observed, the elastic modulus increases with the agarose content and although not shown here, G′ > G′′ at temperatures lower than the melting temperature of the gel, as previously reported for Chi/Aga hydrogels [27].

The melting temperature (*T*_m_) of Chi/Aga-1+Fe5%, Chi/Aga-1.5+Fe5% and Chi/Aga-2+Fe5% ferrogels is 48.2 ± 0.9 °C, 48.4 ± 0.6 °C and 49.5 ± 0.9 °C, respectively. These *T*_m_ values are lower than those previously reported for blank composite Chi/Aga hydrogels, which was 57 ± 1 °C for Chi/Aga-1 and 62 ± 1 °C for Chi/Aga-2 hydrogels [27]. This might be attributed to a lower degree of crosslinking of the agarose in the presence of ferrofluid, which might indicate that the encapsulation of magnetic nanoparticles within composite Chi/Aga hydrogels prevents to some extent the agarose gelation. This is reinforced by the fact that the G′ values obtained at temperatures below *T*_m_ are lower for Chi/Aga ferrogels than those corresponding to blank Chi/Aga hydrogels as can be observed in the inset of Figure 4. 

### 2.4. Specific Power Absorption Experiments

**Figure 5:**
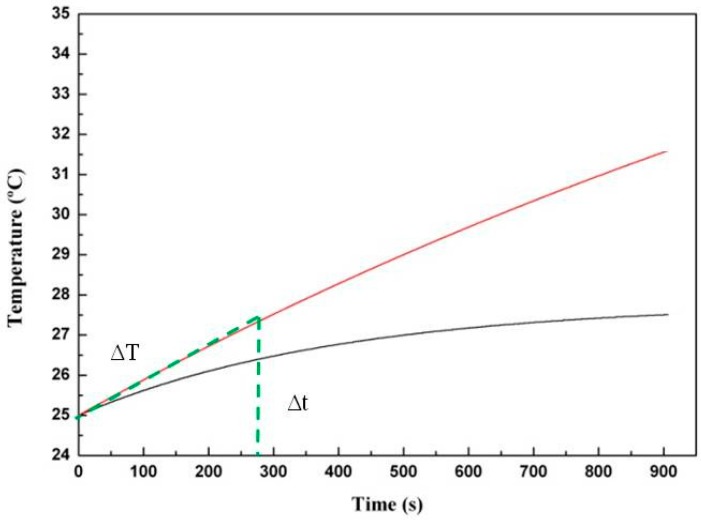
Representative heating performance of Chi/Aga-1.5 ferrogels loaded with ferrofluid (2% (*w*/*v*) (black line) and 5% (*w*/*v*) (red line)), measured at *f* = 418.5 kHz and 24 kA/m.

Figure 5 shows the heating performance of Chi/Aga-1.5 loaded with two concentrations of ferrofluid (2% and 5% (*w*/*v*)), when the samples are submitted to an alternating magnetic field (AMF). As can be observed, there is an increase of temperature with time as a function of the ferrofluid content, being a temperature increase of (Δ*T*) ~2.5 °C for Chi/Aga-1.5+Fe5% and ~1.5 °C for Chi/Aga-1.5+Fe2% after having been subjected to an alternating magnetic field for 900 s.

Specific Power Absorption (SPA) measures the capacity of heat generation of Chi/Aga ferrogels when submitted to an alternating magnetic field. The SPA of the ferrogels was determined from the initial slope of the temperature *vs.* time curves (green part of Figure 5). The temperature increase (Δ*T*) of a given mass of ferrofluid diluted in the system corresponds to that of the selected time interval (Δ*t*), in our case 275 s. SPA was calculated by the following equation [30]:

(1) $$SPA=\frac{m_{LIQ}\;c_{LIQ}+m_{NP}\;c_{NP}}{m_{NP}\;}\;\left(\frac{\Delta T}{\Delta t}\right)$$

where c_LIQ_ and c_NP_ are the specific heat capacities of the liquid carrier and the nanoparticles, respectively. Due to the low concentration of the magnetic material in the gels the following approximation $\;m_{LIQ}c_{LIQ}+m_{NP}c_{NP}\approx m_{LIQ}c_{LIQ}$ can be applied, so that the equation to calculate SPA becomes:

(2) $$SPA=\frac{m_{LIQ}\;c_{LIQ}}{m_{NP}\;}\;\left(\frac{\Delta T}{\Delta t}\right)$$

The SPA values listed in Table 1 show similar values of SPA for both ferrogels. The differences in the slopes (∆*T*/∆*t*) corresponding to the ferrofluid and the ferrogels are attributed to the low amount of magnetic material that is present in the ferrogels. The results corresponding to SPA are normalized to the concentration of magnetic material so that the differences found with respect to the ferrofluid might be due to differences in the agglomeration of the magnetic material in the ferrogels, which might induce a decrease in their SPA [31,32]. 

**Table 1:** Specific power absorption of the samples under study.

Samples	∆*T*/∆*t* (°C/s)	Water content (%)	Fe_3_O_4_ (mg/mL) *	SPA (W/g)
Ferrofluid	1.61	89.7	69.9	96
Chi/Aga-1.5+ Fe2%	0.007	96.8	0.96	30
Chi/Aga-1.5+ Fe5%	0.009	97.1	1.16	32

* For the ferrofluid, Fe_3_O_4_ (mg/mL) is calculated through UV-vis. In the gels, Fe_3_O_4_ (mg/mL) was calculated from the residue values obtained from TGA (Figure 3) considering the water content of the samples and the volume of the cell in the magnetic hyperthermia equipment (1 mL).

## 3. Conclusions 

We report on a simple method to obtain composite ferrogels from the combination of two natural polymers, chitosan and agarose. Agarose gelation in the presence of a mixed aqueous solution containing a dispersion of magnetic nanoparticles and chitosan allows the encapsulation of magnetic nanoparticles within the composite matrixes. The resulting ferrogels present a porous structure in the micrometer range and an increased thermal stability with respect to the corresponding blank hydrogels. This might be ascribed to the presence of interactions between the magnetic nanoparticles and the polymer composite matrix. Notwithstanding, a decrease in the elastic moduli and melting temperature of the ferrogels is observed with respect to blank hydrogels, which suggests that the encapsulation of magnetic nanoparticles within the Chi/Aga hydrogels prevents to some extent agarose gelation. The application of an alternating magnetic field to Chi/Aga ferrogels leads to an increase of temperature so that these materials could be considered potential candidates to be employed in magnetic hyperthermia treatment, even if the low values of SPA obtained with respect to the SPA value corresponding to the initial ferrofluid suggests agglomeration of the magnetic nanoparticles within the polymer composite matrixes. 

## 4. Experimental Section 

### 4.1. Materials

Chitosan (Chi) employed in this work was isolated from shrimp shell (*Heterocarpus vicarious*) and supplied by Polymers Laboratory, National University, Costa Rica. Chitosan has a deacetylation degree of 65% as determined by potentiometric titration and a molecular weight of 362 kDa as determined by viscosity method (ASTM D 2857). Ultra-low gelling temperature agarose (Aga) (gelling temperature in the range of 8–17 °C and melting point in the range of 40–50 °C) was purchased from SeaPrep (Lonza, Switzerland, www.lonza.com). Oleic-acid-coated iron oxide nanoparticles dispersed in water as a ferrofluid (density = 1.08 g/mL), NGAP FeO-05#4, were provided by Nanogap Subnmparticles, Spain. According to the manufacturer, the crystalline form is magnetite, Fe_3_O_4_ and the average size of nanoparticles is 18.55 ± 2 nm. The solid content was 10.30% *w*/*w*. The magnetite concentration in the ferrofluid (Fe_3_O_4_ (mg/mL)), was determined through UV spectroscopy. The ferrofluid was digested in HNO_3_ and HCl 6 M and iron concentration was measured spectrophotometrically at the λ_max_ of 478 nm.

### 4.2. Preparation of Composite Chi/Aga Blank Hydrogels and Chi/Aga Ferrogels

For the preparation of composite Chi/Aga blank hydrogels, different amounts of agarose were dissolved at 60 °C in a 0.5% (*w*/*v*) aqueous chitosan solutions containing 1.0 % (*v*/*v*) of acetic acid. The concentrations of agarose in the mixed solutions were 1.0, 1.5 and 2.0% (*w*/*v*). Then, mixed solutions were poured in Teflon molds and maintained overnight at 4 °C to allow the mixtures to gel. Blank hydrogels were designated as Chi/Aga-1, Chi/Aga-1.5 and Chi/Aga-2. 

Chi/Aga ferrogels were prepared by mixing the ferrofluid solutions (with an initial concentration of 2.0% or 5.0% (*w*/*v*)) with the corresponding Chi/Aga solutions under vortex agitation in a volume ratio of 6:1, until getting homogeneous samples. Then, mixed solutions were poured in Teflon molds and maintained overnight at 4 °C to allow the mixtures to gel. Ferrogels were designated as Chi/Aga+Fex, where x corresponds to the initial ferrofluid concentration, 2.0% or 5.0% (*w*/*v*). 

The water content was determined by weighing the fully hydrated sample (*m*_h_) and the dried sample obtained by freeze-drying (*m*_0_) so that water content (%)= (*m*_h_ − *m*_0_)/*m*_0_.

### 4.3. Scanning Electron Microscopy (SEM)

The surface and cross section of all the samples under study were examined by scanning Electron Microscopy (SEM) (XL30 ESEM, Philips). All the gels under study were freeze-dried. Subsequently, the dried samples were coated with an ultrathin coating of gold deposited on the sample by high-vacuum metallization. Analysis of the pore structure was done using Image Pro 5.0 (media Cybernetics, Maryland, USA) software. 

### 4.4. Thermogravimetric Analysis

The thermal stability of composite Chi/Aga macrogels was evaluated by thermogravimetric analysis (TGA) performed on a Q500 TA Instruments TGA, using a nitrogen stream as purge gas, at a heating rate of 10 °C/min in the range of temperature of 25–700 °C. The experiments were carried out on freeze-dried samples.

### 4.5. Dynamic Oscillatory Measurements

Viscoelastic properties were determined in an AR-G2 rheometer (TA Instruments, Delaware, USA) using 20 mm-diameter steel parallel plates. Temperature sweeps were performed from 5 to 80 °C at 10 °C/min and at 1 Hz frequency. All the experiments were carried out at a fixed torque in the linear viscoelastic regime. The linear viscoelastic region was located with the aid of a torque sweep. All the viscoelastic measurements were performed on hydrogels swelled to equilibrium.

### 4.6. Determination of Magnetic Remote Heating

The specific power absorption (SPA) of Chi/Aga-1.5 ferrogels loaded with two concentrations of ferrofluid (2.0% and 5.0% *w*/*v*) was determined from measurements performed in a commercial AC field applicator (DM100 by nB nanoscale Biomagnetics, Spain) working at ƒ = 418.5 kHz and 24 kA/m (≈300 Oe). Experiments were carried out within a thermally-insulated working space of about 1 cm^3^, using a closed container of 1.0 mL volume. Samples for measurement were prepared directly in the vial. 
